# Role of T cells and cytokines in the pathogenesis of rheumatoid arthritis

**DOI:** 10.1016/j.bbrep.2025.102278

**Published:** 2025-10-07

**Authors:** Monisha Anandan, J. Narayanan

**Affiliations:** Department of Pharmacology, SRM College of Pharmacy, SRM Institute of Science and Technology, Kattankulathur, Chengalpattu, Chennai, India

**Keywords:** Rheumatoid arthritis, T cell subsets, Inflammatory cytokines, T cell-targeted therapy, Biomarkers

## Abstract

Rheumatoid arthritis (RA) is a long-term autoimmune disease. It causes persistent joint inflammation, aberrant tissue growth, and progressive joint deterioration. T cells have a key role in the onset of RA. They identify and trigger other immune cells, and produce inflammatory signals such as interleukin-6 (IL-6), interleukin-17 (IL-17), and tumor necrosis factor-α (TNF-α). Prolonged inflammation and tissue damage are caused by an imbalance between regulatory T cells (Tregs) and pro-inflammatory T helper 17 (Th17) cells. Additionally, the condition is exacerbated by uncontrolled cytokine networks and autoantibody formation. This study highlights the significance of biomarkers, cytokine signaling, and the imbalance of T cell subsets in RA for treatment. Along with updates from clinical trials, we also discuss T cell-focused therapeutics, PD-1/PD-L1 regulation, and Treg adoptive therapy. Finally, we address significant issues and RA's future prospects.

## Highlights

• Autoantigens activate CD4^+^ T cells, releasing IL-17 and TNF-alpha, causing joint inflammation.
• Cytokines attract immune cells, promoting pannus formation and bone damage.
• Dysregulated Th1, Th2, Th9, and Th17 subsets lead to chronic inflammation.
• Dysfunctional Tregs fail to control immune responses, cause autoimmunity in RA.
• Activated T cells stimulate B cells to produce auto antibodies.

## 1 Introduction

Rheumatoid arthritis (RA) is a chronic inflammatory autoimmune illness. It damages joints, results in synovitis, and makes people more disabled. It affects about 0.24 % of persons globally [1,2]. The disease mostly affects synovial joints, especially those in the hands and feet. This causes discomfort, exhaustion, and a decrease in day-to-day functioning [3]. Nearly 22.7 million people were diagnosed with rheumatoid arthritis between 2013 and 2015; more women than men were afflicted. This number is going to increase to 78.4 million by 2040 [4].

T lymphocytes (T cells), macrophages, and fibroblasts are among the immune cells that interact intricately to cause rheumatoid arthritis. TNF-α and IL-1β are two cytokines which promote the persistence of chronic inflammation [5]. Disease progression is significantly influenced by the imbalance between pro-inflammatory and anti-inflammatory cytokines as well as the disruption of T cell subtypes including Th17 and regulatory T cells (Tregs). T cells play a crucial function, and altering their activity may lead to novel therapeutic alternatives [6]. Conventional synthetic disease-modifying antirheumatic drugs (csDMARDs) like methotrexate, hydroxychloroquine, and sulfasalazine are often used as first-line therapy due to their effectiveness and affordability. Patient outcomes have improved with biologic DMARDs (bDMARDs), which include cytokine inhibitors and monoclonal antibodies that target TNF-α, IL-6, IL-1β, IL-17, and Janus kinases [[7], [8], [9], [10]]. However, many individuals do not respond well, and the expensive costs together with significant side effects limit their broad use. These problems highlight a serious research gap: little is known about alternative immunological pathways, particularly those involving T cells. Furthermore, the lack of comprehensive clinical validation for T cell-related markers, such as soluble programmed death protein-1 (sPD-1), restricts their therapeutic efficacy [11].

Chimeric antigen receptor T cells (CART) and regulatory T cells (Tregs), two recent therapeutic advances, give efficient means of directly altering immune responses in rheumatoid arthritis (RA) [12]. T cell subsets, cytokine signaling, and the emergence of biomarkers in RA and developments in clinical trials are summarized in the present study.

## 2 T cell subsets in RA

**Fig. 1:**
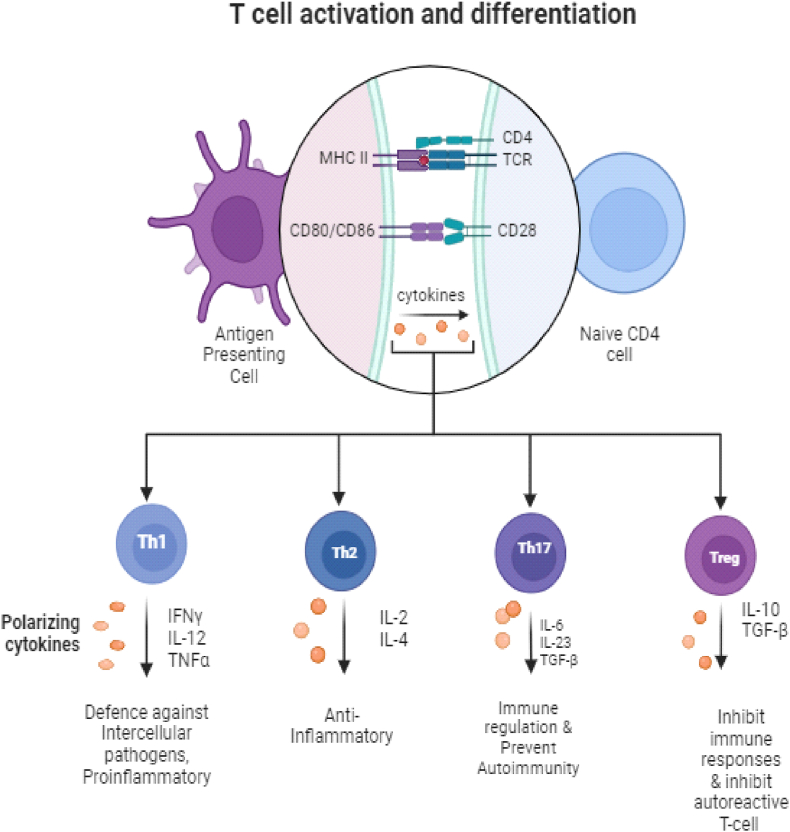
Shows the t cell activation, differentiation, and subsets in rheumatoid arthritis.

T cells develop in the thymus. They are mainly divided into CD8^+^ cytotoxic T cells and CD4^+^ helper T cells. CD4^+^ helper T cells can further split into subsets like Th1, Th2, Th9, Th17, Tfh, and regulatory T cells (Tregs). Each subset has its own unique cytokine profile [13]. Regulatory T cells (Tregs) are crucial for keeping the immune system balanced and for self-tolerance. They use special suppressive methods to control both adaptive and innate immune responses [14]. Naïve CD4^+^ T cells differentiate into subsets depending on the cytokine environment: IFN-γ and IL-12 promote Th1; IL-4 induces Th2; IL-23, IL-6, and TGF-β promote Th17; L-6 induces differentiation into Tfh and IL-2 with TGF-β promotes Foxp3+ Treg [[15], [16], [17]] (Fig. 1).

Regulatory T cells (Tregs) originate from the thymus (thymic Tregs), are generated in peripheral tissues (peripheral Tregs), and can also be induced (induced Tregs). They play a crucial role in regulating the immune system and maintaining peripheral tolerance by inhibiting autoreactive subsets of T cells. tTregs primarily recognize self-antigens in the thymus, while pTregs develop peripherally in response to foreign antigens. iTregs, in contrast, are generated from naïve CD4^+^ T cells under tolerogenic conditions [15,18]. Th1 cells are essential for the immune response to intracellular infections and for the facilitation of inflammation. They produce various cytokines, including Granulocyte-macrophage colony-stimulating factor (GM-CSF), lymphotoxin, and tumor necrosis factor (TNF).

Th1 differentiation is impacted by T cell receptor signals, IL-2/STAT5, IL-12/STAT4, and IFN-γ/STAT1 signaling. The characteristic cytokine of the Th1 subset, IFN-γ, has been associated with autoimmune diseases including rheumatoid arthritis, multiple sclerosis, and type 1 diabetes. Its function encompasses upregulating TLR expression, encouraging immunoglobulin G type changing, and triggering the release of chemokines [19]. IL-4, IL-5, IL-9, IL-10, and IL-13 are secreted by Th2 cells which promote the development of IgE and strong Ab responses, including IgG4 and IgE synthesis and are responsible for allergies and asthma in addition to boosting antibody responses and providing protection against helminth infestations. Th2 cells facilitate the maturation of mast cells stimulated by IgE and encourage the differentiation of eosinophils. The differentiation of Th9 cells is governed by specific transcription factors, with cytokines including transforming growth factor β (TGF-β), IL-4, IL-10, and IL-21 being essential to this process. The intricate process of Th9 cell development involves transcription factors, signaling networks, and cytokines. Important elements include IL-4, STAT6, GATA-3, and the TCR signaling pathway, which activates GATA-3, STAT6, and IL-4 while also stimulating CD4^+^ T cells. Th9 cell formation is stimulated by IL-33 and IL-36γ without IL-4 signaling, whereas their differentiation is regulated by the combination of TGF-β and IL-4 [20]. Th17 cells are characterized by their high secretion levels of the pro-inflammatory cytokine IL-17, in addition to producing TNF-α, IL-6, IL-21, IL-22, IL-26, and GM-CSF. Th17 cells differ from Th1 and Th2 cells in that their differentiation necessitates the presence of transforming growth factor β (TGF-β) and interleukin-6 (IL-6), without the need for interferon-gamma (IFN-γ) and interleukin-4 (IL-4). Additionally, interleukin-1 beta (IL-1β) and interleukin-23 (IL-23) are also crucial for the development of Th17 cells. Critical transcription factors for Th17 cell generation include Retinoic acid receptor (RAR)-related orphan receptor-γt (ROR-γt), signal transducer and activator of transcription 3 (STAT3), and SMADs proteins. Deficiency of STAT3 can prevent Th17 cell development and reduce the activation of ROR-γt. Alterations in the equilibrium between Th1 and Th2 responses, which are antagonistic to one another, can lead to immune-mediated disorders. Without causing significant alterations to the immune system, therapeutic benefits may be obtained by focusing on either kind of response. Additional immunotherapeutic intervention is made possible by the discovery of new molecular targets for pharmaceutical development and the notion that regulatory T-cells govern Th1 and Th2 responses [21].

## 3 Th17/Treg balance in RA

**Fig. 2:**
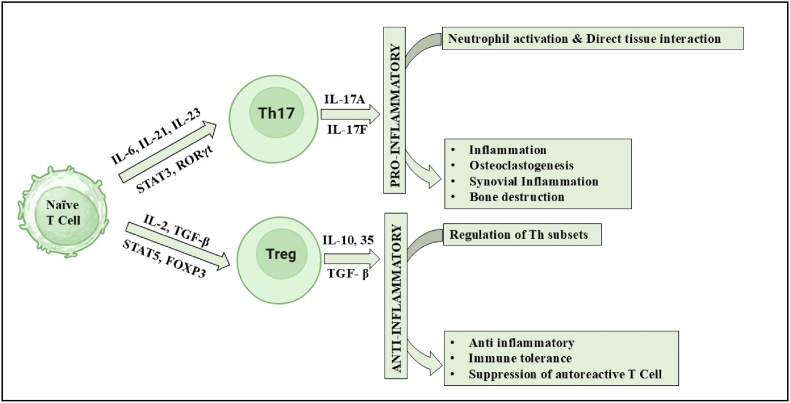
Th17/Treg balance in Rheumatoid Arthritis.

Th17 and Treg cells arise from the same naïve CD4^+^ T cell precursors but diverge under different cytokine conditions. Th17 cells, driven by IL-6, IL-21, and IL-23 through STAT3 and RORγt, produce pro-inflammatory cytokines such as IL-17 that activate synovial fibroblasts, macrophages, chondrocytes, and osteoclasts, joint inflammation and tissue destruction in RA. In contrast, Treg cells, regulated by the TGF-β–SMAD2/3 and IL-2–STAT5–FOXP3 axis, produce anti-inflammatory cytokines and suppress autoreactive T cells, thereby maintaining immune tolerance (Fig. 2). This balance is further modulated by transcriptional regulators: HIF-1α promotes Th17 differentiation while degrading FOXP3, whereas SOCS proteins stabilize Treg identity by fine-tuning IL-2/STAT5 signaling. In RA, a shift toward increased Th17 activity and reduced Treg frequency or function drives persistent autoimmunity and joint damage [22,23].

Evidence from both animal models and human studies highlights the importance of this imbalance. Th17-derived IL-17 not only amplifies inflammation by synergizing with TNFα and IL-1 but also promotes osteoclastogenesis via RANKL upregulation. Although modest enrichment of Th17 cells is observed in synovial fluid, their frequency is variable and relatively low in tissue, suggesting heterogeneity. Other IL-17–producing cells, including mast cells, macrophages, and fibroblasts, may contribute significantly to the IL-17 pool. Moreover, synovial Th17 cells frequently co-express TNFα, enhancing pathogenicity, but show limited IL-22 production and low IL-23R expression, raising questions about their stability in chronic disease. Thus, RA pathogenesis is not driven solely by Th17 expansion but by their cytokine profile, plasticity, and interactions with other immune and stromal cells. The Th17/Treg imbalance contributes both to synovial inflammation and osteoclastic bone resorption, making it an attractive therapeutic axis [24,25].

IL-17A emerged as a promising target, with early phase I/II studies of secukinumab (AIN457) and ixekizumab showing improved ACR20/50 responses. However, subsequent phase II/III trials yielded modest efficacy, with no superiority over abatacept (CTLA-4-Ig), and IL-17RA blockade (brodalumab) or dual TNF/IL-17A inhibition (ABT-122) also failed to show major advantages. In contrast, IL-6 receptor blockade with tocilizumab (TCZ) consistently restored the Th17/Treg balance, even in patients without elevated serum IL-6, and clinical improvement correlated with normalization of this ratio [23]. Collectively, these findings support the concept that therapies modulating Th17/Treg dynamics—by inhibiting Th17 differentiation or enhancing Treg stability—are central to RA management. Given the plasticity of CD4^+^ T cell subsets, interventions such as TCZ may re-establish immune homeostasis, while the Th17/Treg ratio itself may serve as a biomarker for treatment efficacy [26].

## 4 Mechanisms of T cell activation in RA

### 4.1 Dendritic cells

Dendritic cells (DCs), a distinct category of antigen-presenting cells (APCs), are essential for both the initiation and regulation of immune responses [27]. They help T cells grow and preserve immunological tolerance by identifying, processing, and delivering antigens to T cells. The capacity of dendritic cells to regulate the immune system is influenced by their level of maturity. Proinflammatory mediators regulate immature DCs, which suppress the immune system by postponing T cell activation [28]. In normal individuals, they support the preservation of peripheral antigen-specific immunological tolerance. On the other hand, DCs exacerbate inflammatory conditions and damage to end-organs in rheumatic illnesses [29]. DCs are distinct cells that present antigens that may be located in lymph nodes and skin, among other locations. They have the ability to gather, analyse, and present antigens, triggering immunological responses in B cells, CD4+T, and naïve T. Through their interactions with B cells or indirectly induction of CD4^+^ helper T cell proliferation and differentiation, contribute to the regulation of humoral immunity [30]. Dendritic cells travel to lymph nodes, process antigens onto MHC molecules, release cytokines, and direct naïve T cells towards Th-1, Th2, or Th17 lineages, while also facilitating the development of antibody-producing B cells [31].

### 4.2 Macrophage

The innate immune system depends on macrophages and monocytes to perform various functions related to immunity and maintaining homeostasis. The bone marrow produces monocytes, which in inflammatory environments develop into macrophages. The macrophages are diverse cellular entities that regulate distinct tissue reactions. They can be further classified into two subpopulations: M1 and M2. The macrophages are necessary for tissue remodeling, healing, and homeostasis maintenance, whereas monocyte can be distinguished by cell surface markers [32]. Macrophages produced from monocytes and tissue-resident macrophages are the two primary sources of macrophages [33]. Increased synovial cell infiltration has been associated with joint degeneration and inflammation. Decreasing the functioning of the mononuclear phagocyte system is associated with the success of conventional rheumatic therapy. Various functions are played by macrophage markers in RA, including C-X-C motif ligand (CXCL) [34,35].

### 4.3 B cell

In the bone marrow, cytokines and adhesion molecules regulate the maturation and differentiation of B cells [36]. Hematopoietic stem cells found in fetal bone marrow and liver are utilized in the regulated process of B cell development, which produces functional peripheral subsets [37]. Numerous cytokines are released by the B cells, including regulatory B cells (Bregs), which help to control inflammatory reactions and preserve tolerance. Because it promotes the development of T cells that regulate and inhibits cytokines that are proinflammatory [38]. B and T cells are the two key components of the immune system. They are generated in the bone marrow and undergo several developmental stages. Once they exhibit a BCR, they enter the bloodstream and proceed to the spleen. The blood contains young B cells called naive B cells (NBCs). After being activated by an antigen, they reach germinal centres where affinity maturation occurs [39].

**Fig. 3:**
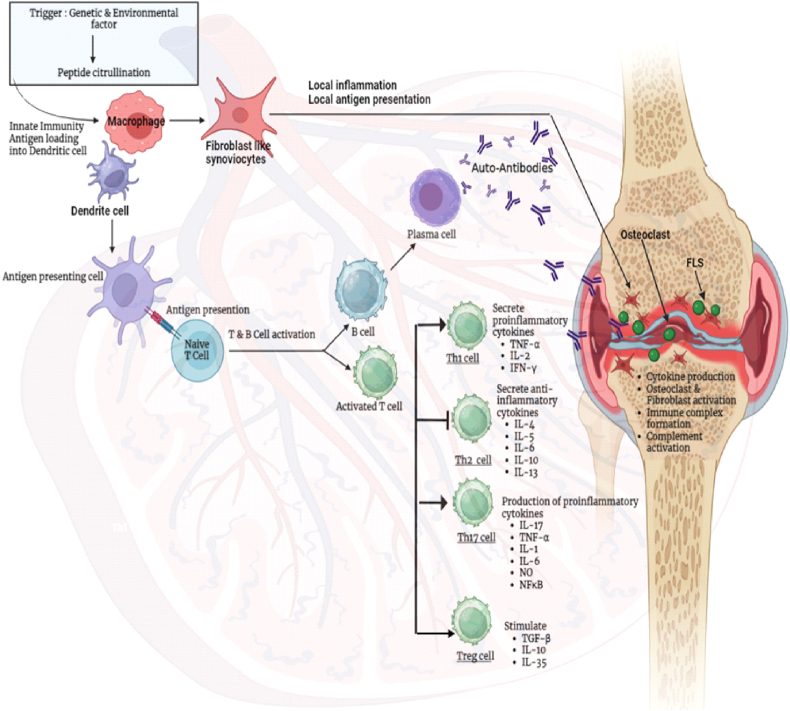
The role of immune cells and cytokines in the development of rheumatoid arthritis.

B cells play a crucial role as antigen-presenting cells in proteoglycan-induced arthritis (PGIA), greatly contributing to the activation of autoreactive T lymphocytes. In rheumatoid arthritis, B cells give their antigens to CD4^+^ T helper cells, categorized as follicular helper cells (Tfh) and peripheral helper cells (Tph). Tfh and Tph cells release CXCL13 and IL-21, which are crucial for B cell development and the generation of autoantibodies. B lymphocytes in the peripheral blood of rheumatoid arthritis patients may release many cytokines, including TNF-α, IFN-γ, IL-6, IL-1β, IL-17, and IL-10, which facilitate bone deterioration (Fig. 3). Regulatory B (Breg) cells, which perform immunosuppressive roles, are responsible for synthesizing anti-inflammatory cytokines like IL-10, TGFβ, and IL-35 [40].

### 4.4 Synovial fibroblasts

Synovial cells, particularly active synovial fibroblasts (SFs), are linked to joint degradation in RA [41]. A big, pale nuclei with noticeable nucleoli, which signifying active RNA metabolism, is a characteristic of rheumatoid arthritis stem cells (RASFs). They have the ability to proliferate in cultured cells and induce aggressive behaviour in nearby cartilage and bone tissues. Inflammation that persists results from these factors' recruitment and accumulation of cells from the immune system in the synovium. The inherent damaging qualities of RASFs are triggered by cytokines such as TNF and IL-1, which also contribute to their aggressive behaviour. A further characteristic of inflammatory synovial cells is blood vessel development, and factors that promote it are expressed by RASFs [42]. Rheumatoid arthritis is largely caused by activated fibroblasts in the synovial fluid (RA-SFs), which react to cytokines that promote inflammation and change molecularly. They adhere to cartilage in joints and invade the matrix of extracellular cells with an aggressive temperament. Research from the severely combination immune deficient rat co-implantation model supports the idea that the activation of them is an innate trait or a reaction to cytokines associated with inflammation [43].

## 5 Biomarkers of T cell

T cells are essential in the therapy of RA, facilitating neoangiogenesis, lymphoid organogenesis, and osteoclastogenesis. Nonetheless, T-cell-targeted therapy must account for the distinctiveness of T-cell production, turnover, and differentiation in affected individuals [44]. Several genetic and biochemical indicators have been associated with therapy in rheumatoid arthritis, and biomarkers can function as impact modifiers. The potential of biomarkers to facilitate early and rapid diagnosis, prognosis, and treatment of rheumatoid arthritis (RA) is delineated here. T-Cell biomarkers in RA are summarized in (Table 1).

**Table 1:** PD-1/PD-L1, FoxP3 and FOXO3a T-Cell biomarkers in RA.

Marker	Author & Year	Immune cell	Regulation in RA	Findings
PD-1/sPD-1	Hassan et al., 2015	T cells, B cells, synovial fluid	Upregulated	Higher plasma & synovial sPD-1 correlated with DAS28 & HAQ, suggesting it as a marker of disease activity.
	Wu et al., 2020	Peripheral blood (RA-ILD patients)	Upregulated	Elevated sPD-L1 linked to interstitial lung disease, correlated with RF, HRCT score, and reduced lung function.
	Smith et al., 2021	Osteoclasts, APCs	Upregulated	PD-L2 co-expressed with RANKL; regulates osteoclast activity and bone metabolism under inflammatory conditions.
FoxP3 (Treg marker)	Abaza et al., 2013	CD4^+^CD25^+^ Tregs	Downregulated	RA patients showed altered FoxP3: Treg ratio (shift from 1:3 in controls to 1:1 in active RA).
	Laragione et al., 2023	FoxP3+ Tregs	Upregulated	High-magnesium diet in mice increased FoxP3+ Tregs & IL-10+ T cells, reduced pro-inflammatory cytokines.
	Al-Zifzaf et al., 2025	FoxP3+CD4^+^CD25+high Tregs	Downregulated	Lower FoxP3 expression impaired Treg suppressive function, promoting autoimmune progression.
FOXO3a	Xu et al., 2021	T cells, dendritic cells	Dysregulated	Controls T cell apoptosis, dendritic cell tolerance, and cytokine regulation; implicated in RA and other autoimmune diseases.

### 5.1 PD-1 (programmed cell death protein 1)

PD-1 (CD279) is a member of the CD28/B7 family of immunological checkpoint receptors that controls T-cell activation. T cells, B cells, myeloid cells, NK cells, and thymic cells all express it. By regulating T-cell proliferation, cytokine production, and co-stimulatory signals, PD-1, in conjunction with its ligands, PD-L1 and PD-L2, influences autoimmunity. While PD-L2 expression is limited to professional antigen-presenting cells (APCs), PD-L1 is widely distributed and preserves peripheral tolerance [45,46]. Mechanistically, Lck facilitates the phosphorylation of PD-1's immunoreceptor tyrosine-based inhibitory motif (ITIM) and immunoreceptor tyrosine-based switch motif (ITSM) motifs upon PD-1 activation in T cells. This gives SHP2 and sometimes SHP1 docking sites, which dephosphorylate important TCR downstream signaling molecules and reduce T-cell activation. By impairing both cerebral and peripheral tolerance, dysregulated PD-1 signaling plays a role in autoimmune disorders [47].

According to Smith et al., 2021, PD-L2, a ligand of PD-1, co-expresses with RANKL upon TNF-α stimulation, controlling osteoclast activity and differentiation. According to this, PD-L2 plays a critical role in bone metabolism, especially in lowering osteoclast activity and stopping bone deterioration in inflammatory settings [48]. Hassan et al., 2015 found significantly elevated serum and synovial levels of soluble PD-1 (sPD-1) in RA patients compared with healthy controls. Increased sPD-1 correlated strongly with DAS28 scores, HAQ scores, and radiographic severity, indicating that sPD-1 may serve as a biomarker of disease activity and progression in RA [49]. Wu et al., 2020 reported that serum sPD-L1 levels were elevated in RA patients with interstitial lung disease (RA-ILD) compared to RA patients without ILD and healthy controls. High sPD-L1 levels correlated negatively with lung function (FVC, DLCO) and positively with rheumatoid factor (RF) and HRCT scores, linking it to RA-ILD progression [50].

### 5.2 Foxp3− CD4^+^ CD25^−^ T

The major regulator of regulatory T cells (Tregs), which are necessary for immunological self-tolerance and homeostasis, is the forkhead box transcription factor (FoxP3). FoxP3 expression is essential for CD4^+^CD25^+^ Treg formation and suppression. Decreased FoxP3 expression affects Treg function and plays a role in the etiology of autoimmune disorders, such as RA [51]. Xu et al., 2021 revealed FOXO3a, a similar transcription factor that belongs to the Forkhead box O family, is important for aging, oxidative stress responses, autophagy, cell proliferation, and apoptosis. FOXO3a modulates cytokine expression, controls T-cell activation, and enhances dendritic cell tolerance. FOXO3a also regulates cytokine expression and participates in intercellular interactions. Recent data demonstrates Numerous autoimmune conditions, such as RA, inflammatory bowel disease, and systemic lupus erythematosus, have been linked to dysregulation of FOXO3a [52].

Abaza et al., 2013 examined the modulation of Tregs in RA patients in comparison to healthy controls. They found that RA patients had considerably larger amounts of CD4^+^CD25^+^ Tregs, although their FoxP3 expression ratios were different. The ratio of FoxP3/FoxP3^+^ cells was approximately 1:3 in healthy controls, 2:3 in remission, and 1:1 in active RA. This imbalance draws attention to functional flaws in RA Tregs. According to the study, restoring Treg function and using FoxP3 expression as a therapeutic approach may be possible. FoxP3 continues to be the most accurate identification despite difficulties brought on by the absence of a single conclusive Treg marker [51]. Laragione et al., 2023 reported that in mice with RA and collagen-induced arthritis, a high-magnesium diet decreased the severity of the arthritis and joint destruction. This intervention reduced the production of pro-inflammatory cytokines while increasing the amount of FoxP3^+^ Tregs and T cells that produce IL-10, indicating that dietary regulation of Tregs could be used as an adjuvant treatment [53]. Al-Zifzaf et al., 2025 investigated FoxP3 expression in CD4^+^CD25^+^high Tregs was shown to be considerably lower in RA patients than in healthy controls. These decreases exacerbated autoimmune reactions by impairing Treg inhibitory activity and encouraging their transformation into effector T cells. The results highlight FoxP3 as a crucial indicator of Treg dysfunction and disease activity in RA [54].

## 6 Inflammatory cytokines

**Fig. 4:**
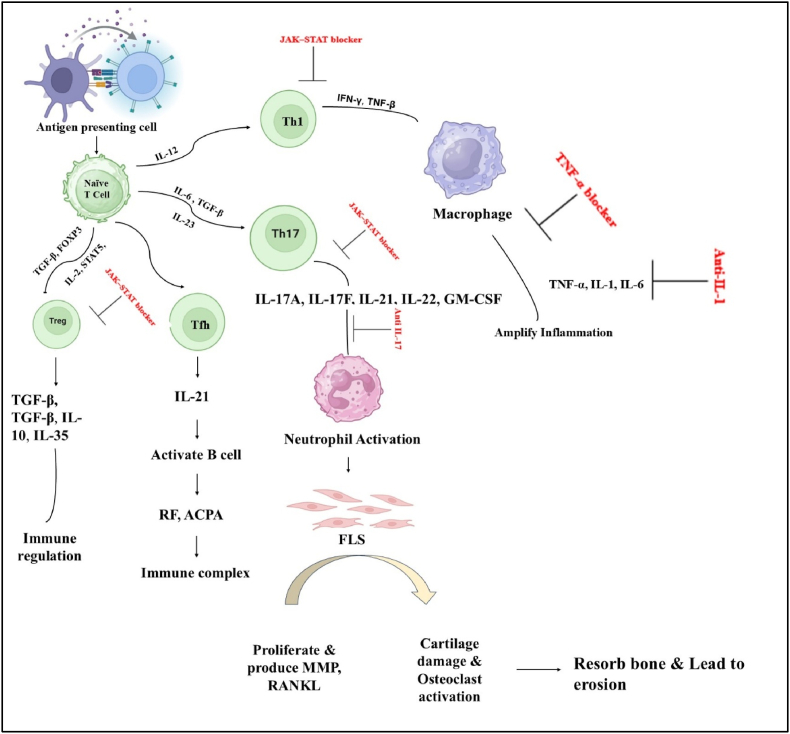
Role of t cell subsets and cytokines in RA inflammation.

Cytokines, which are small proteins secreted by signalling cells regulate leukocyte recruitment, cartilage and bone degradation, and inflammatory and immunological responses. They serve as indicators to track the effectiveness of treatment and distinguish those who are at danger. Monocyte-derived macrophages are the main source of TNF, and synovial macrophages can induce fibroblast growth, inflammation, and injury to the synovium [55] (Fig. 4).

### 6.1 TNF- α

TNF-α, a cytokine produced by macrophages, T cells, natural killer cells, and mast cells, regulates immune responses related to inflammation. It is primarily induced by lipopolysaccharides in gram-negative bacterial membranes [56]. Excess TNF-α is produced by synovial cells in RA [57]. TNF-α binds to TNF receptors I and II (TNF-R), which control important signalling pathways such as NF-κB and MAPK that lead to cell death and pro-inflammatory reactions [58,59]. These pathways control the production of chemokines, osteoclast differentiation, cell proliferation, and apoptosis [60]. TNF-α promotes the inflammatory and immune response by recruiting neutrophils and monocytes to the sites of infection [59]. In RA, TNF-α is a chief mediator of bone damage and synovial hyperplasia [61]. Furthermore, TNF can mediate the inflammatory response by activating the NF-KB signalling pathway and causing the synthesis of inflammatory cytokines like IL-1β, IL-6, etc [62]. Furthermore, TNF-α can be inhibited with monoclonal antibodies including etanercept (ETA), golimumab, adalimumab, and infliximab (IFX), directly target TNF-α by blocking the TNFR pathway, which lowers disease activity and structural damage [63,64]. Experimental data also show that Galectin-9 regulates TNF-α signalling: Gal-9 levels are elevated in RA synovium but decrease following TNF-α inhibitor therapy, suggesting a modulatory role [65]. Furthermore, IL-37 has been shown to suppress TNF-α–induced pyroptosis in RA fibroblast-like synoviocytes (RA-FLSs) by blocking the NF-κB/GSDMD pathway, highlighting potential adjunct therapeutic strategies [66].

### 6.2 IL-33

The cytokine family IL-1 includes IL-33, which is available in two forms immature (iIL-33) and mature (mIL-33). It is released in small quantities by living cells and controls inflammation and immune responses. By stimulating a number of biochemical processes in macrophages, mast cells, granulocytes, and other cells, IL-33 plays a critical role in immunological disorders like RA. High quantities of IL-33 have been found in RA patients' serum, synovial fluid, and inflammatory lesions, indicating a role for the protein in the pathophysiology of the condition [67]. IL-33 signals through its receptor ST2, activating MyD88, IRAK1/4, and TRAF6, which in turn trigger MAPK, NF-κB, and AP-1 pathways. These cascades promote Th2-related cytokine production and recruit neutrophils, contributing to chronic inflammation in RA. IL-33 also influences macrophage polarization, shifting the balance between pro-inflammatory M1 and anti-inflammatory M2 subsets, thereby altering cytokine networks that drive RA progression [68]. Clinically, IL-33 exacerbates arthritis in collagen-induced models, while blockade of the IL-33/ST2 axis with soluble ST2 or neutralizing antibodies reduces joint inflammation and damage. These findings highlight the IL-33/ST2 pathway as a promising therapeutic target in RA [69,70].

### 6.3 IL-6

IL-6, a pleiotropic cytokine, plays a crucial role in both innate and adaptive immunity. It is produced by macrophages and other cell types, influencing neutrophil and mononuclear cell infiltration, CD4^+^ T-cell growth, and various illnesses [71]. IL-6 signals through two distinct pathways. Classical signalling, mediated by membrane-bound IL-6R and gp130, supports homeostatic and regenerative processes with predominantly anti-inflammatory effects. In contrast, transsignalling, via soluble IL-6R, drives chronic inflammation by activating JAK/STAT and MAPK cascades, leading to phosphorylation of STAT3, ERK, and Akt. These events enhance T helper cell differentiation, B-cell maturation into plasma cells, autoantibody production, and angiogenesis [72,73]. Serum and synovial fluid levels of IL-6 are increased in rheumatoid arthritis (RA), where it plays a role in pannus development, synovial angiogenesis, and joint destruction. By triggering acute-phase reactants such C-reactive protein, fibrinogen, and serum amyloid A, it increases systemic inflammation and encourages osteoclast activation, which results in bone degradation [74]. Additionally, IL-6 works in conjunction with TNF-α and IL-1β to increase the production of VEGF, which promotes pannus vascularization [75]. Accordingly, IL-6 is regarded as a key mediator of RA pathogenesis in clinical settings, and IL-6 receptor inhibitors, such tocilizumab and sarilumab, have shown notable effectiveness in lowering inflammation and averting structural damage [76].

### 6.4 GM-CSF

Granulocyte-macrophage colony-stimulating factor (GM-CSF) is a versatile cytokine that regulates blood cell formation and immune cell activation. It connects innate and adaptive immunity [77]. GM-CSF is produced by various cells, including T and B cells, monocytes, fibroblasts, neutrophils, and tumor cells, and is stimulated by factors like TNF and IL-1 [78].

The GM-CSF receptor (GM-CSFR) has a ligand-specific α subunit and a common β subunit (βc). It shares the βc subunit with the IL-3 and IL-5 receptors. The βc subunit is pre-associated with Janus kinase 2 (JAK2) and acts as the main signalling component. When GM-CSF binds, the receptor forms a larger dodecamer complex. This complex enables dimerization, βc transphosphorylation, and starts the signalling process. Structural studies show that site 4 is important for activating the GM-CSF and IL-3 receptors. Once activated, the GM-CSFR triggers JAK2/STAT5 signalling and engages the MEK/ERK, PI3K/Akt, and NF-κB pathways. A key regulator downstream is interferon regulatory factor 4 (IRF4), which promotes dendritic cell–like features in GM-CSF–stimulated monocyte precursors. Upregulating IRF4 leads to pro-inflammatory macrophage polarization and improves antigen-presenting capabilities, which increases inflammatory responses [77,79,80]. GM-CSF has become a promising treatment target for rheumatoid arthritis (RA) and other autoimmune inflammatory diseases. Recent clinical trials with otilimab, a monoclonal antibody that blocks GM-CSF, show its potential. Although a phase 2b trial failed to meet its main goal of DAS28-CRP remission, it revealed a significant 30 % improvement in clinical outcomes compared to placebo. Proof-of-concept studies showed lower levels of serum CCL17 and meaningful improvements in pain and hand function. This suggests that blocking GM-CSF may affect both inflammation and pain related to RA. However, safety concerns still exist. GM-CSF inhibitors influence the JAK/STAT pathway, which increases the risk of herpes zoster reactivation. They may also weaken vaccine responses or increase the chance of developing autoimmune conditions like colitis and type 1 diabetes. On the positive side, GM-CSF blockade has not been linked to a higher risk of cancer, lung disease, or heart issues in safety analyses of mavrilimumab trials. Notably, GM-CSF might also have unexpected protective effects in the lungs by slowing interstitial fibrosis, suggesting its role can vary depending on the tissue. Inhibiting GM-CSF offers a new treatment strategy for RA, with promising results in managing pain and improving function, but it needs careful long-term monitoring for infections and autoimmune reactions [81].

### 6.5 IL-17

Interleukin-17 (IL-17) is a pro-inflammatory cytokine involved in the pathogenesis of RA [67]. The IL-17 family includes six cytokines (IL-17A, IL-17B, IL-17C, IL-17D, IL-17E, and IL-17F) and five receptors. Although IL-17A can be produced by CD8^+^ T cells, γδ T cells, and NKT cells, its main source is CD4^+^ Th17 cells. In RA, both synovial tissue and synovial fluid exhibit increased levels of IL-17 [82]. IL-17 exerts its effects through IL-17 receptor (IL-17R) binding, which triggers phosphorylation cascades involving NF-κB and MAPK signaling [83]. This leads to the production of pro-inflammatory cytokines and chemokines that amplify joint inflammation. In fibroblast-like synoviocytes (RA-FLS), IL-17 enhances secretion of vascular endothelial growth factor (VEGF), promoting angiogenesis and synovial vascularization in early disease [84]. It also induces matrix metalloproteinases (MMPs), driving extracellular matrix breakdown in cartilage and synovium, and can trigger autophagy in RA-FLS, leading to mitochondrial dysfunction [85]. Through NF-κB activation, IL-17 also promotes osteoclastogenesis and pannus formation, contributing to cartilage and bone destruction in RA. Its central roles in angiogenesis, tissue damage, and chronic inflammation make IL-17 and its receptor important therapeutic targets. Results from IL-17 inhibitor trials in RA have been inconsistent. In phase II and proof-of-concept studies, secukinumab showed early signals of efficacy (ACR20 and DAS28-CRP improvement) but did not consistently meet primary endpoints. Ixekizumab demonstrated dose-dependent responses in biologic-naïve patients, though effects were less pronounced in TNF-IR patients. In contrast, the anti-IL-17RA antibody brodalumab showed no meaningful benefit, with ACR responses similar to placebo. Overall, IL-17 blockade is highly effective in psoriasis, psoriatic arthritis, and ankylosing spondylitis, but its role in RA remains unclear. Future progress will likely depend on patient stratification and biomarker-driven precision medicine [86].

### 6.6 IL-21

IL-21 is a type I cytokine primarily produced by activated CD4^+^ T cells, particularly T follicular helper (Tfh), Th17, and natural killer T (NKT) cells [87,88]. T and B cell development, proliferation, and function are all impacted by the cytokine IL-21, which controls immune cell activity. Additionally, it encourages CD8 + T and NK cell development and cytotoxicity. In addition to promoting or suppressing immune responses and promoting the development of memory and naive B cells into plasma cells, IL-21 has been connected to a number of illnesses, including autoimmune diseases, cancer, and allergies [89]. This cytokine binds to the IL-21 receptor (IL-21Rγ) found on Th17 cells, Tfh cells, fibroblast-like synoviocytes (FLS), and macrophages. This binding activates various signalling pathways like JAK/STAT, MAPK, PI3K/Akt Pathway that control survival, growth, and inflammatory responses. IL-21 activates JAK1/STAT3 signalling. This drives Th17 differentiation and increases IL-17 production. It also promotes B-cell growth and antibody production. When IL-21R is blocked, STAT3 activation decreases. This leads to lower levels of inflammatory cytokines and autoantibody production in experimental arthritis models. IL-21 stimulates ERK1/2 and p38 MAPK signalling in RA-FLS and macrophages. This boosts cell growth, cytokine release (such as TNFα and IL-6), and MMP expression. As a result, it contributes to synovial hyperplasia and joint damage. IL-21 activates PI3K/Akt signalling in the RA synovium. This promotes the survival of FLS, macrophages, and osteoclast precursors, worsening bone erosion and cartilage breakdown. Blocking PI3K reduces the FLS growth and cytokine release driven by IL-21 [90,91]. In rheumatoid arthritis, high plasma IL-21 levels connect to greater disease activity. Experimental models indicate that blocking IL-21 signalling lowers pro-inflammatory cytokine production and slows down the onset of arthritis. In patients, targeting IL-21 and IL-21R has shown only slight improvements. However, JAK inhibitors, which stop IL-21's downstream signalling, have proven effective in reducing inflammation and the severity of the disease. Therefore, while directly blocking IL-21 is still being studied, inhibiting it signalling pathways presents a hopeful treatment option for rheumatoid arthritis [92].

## 7 Therapeutic targeting in RA

**Fig. 5:**
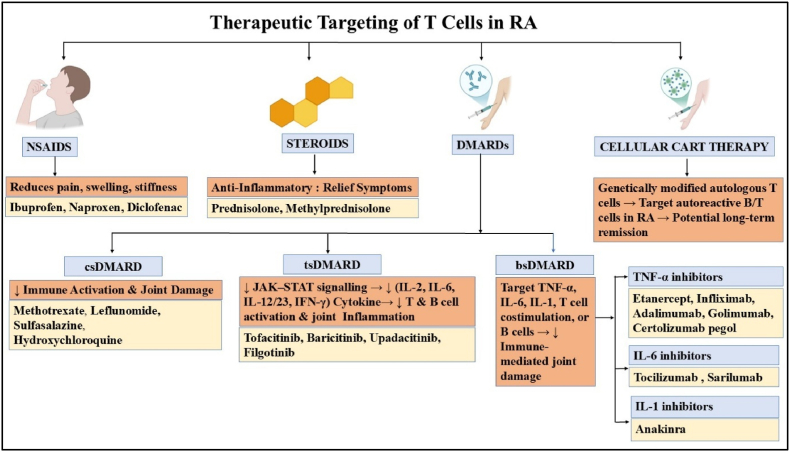
Drugs and cellular therapies targeting t cells and cytokines in rheumatoid arthritis.

While NSAIDs and corticosteroids are important for relieving symptoms in RA, they mainly focus on controlling inflammation and do not specifically target the immune pathways that drive the disease. In contrast, disease-modifying antirheumatic drugs (DMARDs) provide benefits by targeting the activation of immune cells, especially T cells and their cytokine signalling. csDMARDs, such as Methotrexate, hydroxychloroquine, leflunomide, and sulfasalazine, directly impact T cell antigen presentation, pyrimidine synthesis, and cytokine production, which reduces inflammation driven by T and B cells. Targeted synthetic DMARDs (tsDMARDs), like tofacitinib, enhance T cell therapy by blocking JAK-mediated cytokine signalling. Biological DMARDs (bDMARDs) include monoclonal antibodies and fusion proteins that inhibit T cell-related cytokines like TNF-α and IL-6 or deplete B cells that interact with T cells (Fig. 5). Despite the advances, significant challenges persist: incomplete responses in numerous patients, infections related to treatment, inconsistent persistence, and specific toxicities associated with drugs. These constraints have showed interest in next-generation immunotherapies, such as chimeric antigen receptor (CAR)–T cells and various cellular strategies aimed at selectively targeting and eliminating pathogenic T cell subsets while preserving protective immunity [93].

### 7.1 DMARDS

Disease-modifying anti-rheumatoid drugs reduce joint damage. DMARDS are classed as conventional, targeted, and biological drugs.

#### 7.1.1 Conventional synthetic DMARD (csDMARD)

Conventional Synthetic traditional DMARDs such as methotrexate, hydro chloroquine, and Sulfasalazine are often used to treat RA. For many years, these drugs have been used to treat diseases and prevent joint damage, either separately or with glucocorticoids or biologics.

##### 7.1.1.1 Methotrexate

Methotrexate is anti-folate, first-line drugs used together with glucocorticoids to alleviate joint inflammation [94]. The antiproliferative and anti-inflammatory actions of MTX are regulated by genetic diversity in the proteins, which changes the drug's kinetic profile [95]. MTX regulates the immune system, immunological cells, and inflammatory cells in synovium infiltration by lowering proinflammatory cytokines. DNA, RNA, and protein synthesis are inhibited by blocking the development of dihydrofolate reductase and thymidylate synthase while restricting purine and pyrimidine synthesis. It exerts anti-inflammatory effects via the adenosine path, inhibiting inflammation and restoring neutrophils [96]. The MTX dosage for treating RA is 10–25 mg per week, which has been used for over 30 years to reduce symptoms such as joint damage. It is also used to treat other conditions such as psoriatic arthritis, dermatomyositis, and systemic lupus erythematosus, despite the fact that it has many side effects such as hematologic, malignant, hepatic, nausea, pulmonary, infectious, mucocutaneous, renal, neuro-psychiatric, and musculoskeletal. In the 1990s, anaemia, leukopenia, and thrombocytopenia were recorded in 3–10 % of persons [97].

##### 7.1.1.2 Hydroxychloroquine (HCQ)

Hydroxychloroquine is an antimalarial drug that has been used in rheumatoid arthritis for decades. HCQ is a structural derivative of chloroquine; the addition of a side chain containing the hydroxyl group of chloroquine has fewer negative effects, yet it is used to treat autoimmune illnesses. Hydroxychloroquine can cause gastrointestinal discomfort, allergic responses, neurotoxicity, skin pigmentation, renal toxicity, and cardiomyopathy [98]. HCQ decreases proinflammatory cytokines transcription and inhibits autophagy also interfere with antigen presenting cell and lysosomal activity. Being a lipophilic weak base drug that stores in lysosomes and raises pH, it crosses the cell membrane with great ease. This change in pH causes lysosomal protease, reduces antigen binding to the α and β chains of MHC class II molecules, and causes certain disruptions in the intracellular processing of antigens. HCQ preferentially inhibits autoimmunity but does not compromise immunisation against foreign antigens since autoantigens generally have a lower affinity for MHC class II molecules than non-self-antigens [99].

##### 7.1.1.3 Sulfasalazine (SSZ)

Sulfasalazine is now commonly prescribed as a second-line medication (DMARD) to treat rheumatoid arthritis [100]. As a salicylate derivative of amino acids with anti-inflammatory, immunomodulatory, and antiproliferative properties, sulfasalazine is used to treat RA and ulcerative colitis. By use of an azo bond, sulfasalazine is covalently bonded to mesalazine and sulfonamide. Its 30 % bioavailability in the small intestine is absorbed there. Mesalazine and sulfapyridine are released in the large intestine through azoreduction by bacterial azoreductases. It dispersed and absorbed throughout the body without crossing the blood-brain barrier. The salicylate component functions as an anti-inflammatory drug, and the sulfa moiety possesses antibacterial qualities [101]. The common side effects of sulfasalazine are skin rashes, nausea, macrocytosis, dyspepsia and abdominal discomfort [102].

#### 7.1.2 Targeted synthetic DMARDs

##### 7.1.2.1 JAK STAT inhibitors

Janus kinase (JAK) inhibitors are a class of recently developed targeted synthetic DMARDs for rheumatoid arthritis (RA). Unlike biologic therapies, which act extracellularly, these small molecules are orally administered and inhibit the intracellular JAK–STAT signalling pathway, a key regulator of cytokine-driven inflammation in RA. By modulating multiple pro-inflammatory cytokines, including IL-6, IL-2, IFNγ, and GM-CSF, JAK inhibitors indirectly regulate T-cell activation, proliferation, and differentiation, providing a multi-cytokine, T-cell-directed therapeutic approach compared with single-target biologics. The initial approved JAK inhibitors such as tofacitinib, baricitinib, and Upadacitinib demonstrate clinical efficacy comparable to TNF inhibitors, with the added benefit of convenient oral administration, which aligns with patient preferences over parenteral biologics [103].

###### a Tofacitinib (TFB)

Tofacitinib selectively inhibits JAK1 and JAK3, modulating T-cell-dependent cytokine pathways. Long-term use is limited by adverse effects due to the broad role of JAK/STAT in haematopoiesis and immunity. FDA boxed warnings highlight increased risks of thrombosis and mortality at higher doses, underscoring the need for safety optimization [104]. In RA, oral tofacitinib (5 mg twice daily in the EU) is effective as monotherapy or with csDMARDs, significantly reducing disease activity and improving quality of life over long-term use (up to 96 months). Most adverse events are infections, including herpes zoster, generally manageable. When combined with methotrexate, tofacitinib is non-inferior to adalimumab in efficacy, with comparable tolerability. This supports its role as a T-cell and cytokine-directed oral DMARD option [105].

###### b Baricitinib

Baricitinib is an oral, selective JAK1/JAK2 inhibitor with lower potency for JAK3, modulating pro-inflammatory cytokines (IL-6, TNF-α, IL-8, IFN-α/γ, GM-CSF) and indirectly regulating T-cell activation and cytokine-mediated inflammation in RA [106]. Rapidly absorbed reached Tmax in 1–1.5 h, high bioavailability of 79 %, moderate tissue distribution, 50 % protein binding, minimal hepatic metabolism of 10 %, and primarily renally excreted by 75 % unchanged [107]. Approved 4 mg once daily for moderate-to-severe RA after methotrexate or TNF inhibitor failure. Can be used as monotherapy or with methotrexate. Trials and real-world data show rapid improvements in disease activity, inflammatory markers, and patient-reported outcomes. Common adverse events include infections notably herpes zoster, hyperlipidaemia, anaemia, and mild creatinine increases. Serious events such as malignancy, TB, thrombosis are rare but monitored. With oral convenience and rapid onset. Limitations include infection risk and variability in long-term real-world outcomes. Experimental therapies like CAR-T may complement JAK inhibition in the future [108].

###### c Upadacitinib

Upadacitinib is a selective JAK1 inhibitor with 40- to 190-fold selectivity over other JAK isoforms, allowing potent inhibition of JAK1-dependent cytokine signalling (IL-6, IL-2, IFNγ) while sparing JAK2-mediated hematopoietic pathways. This selectivity reduces off-target effects on erythropoiesis and NK cells [109]. Phase I/II studies and the Phase III SELECT program demonstrated rapid, dose-dependent improvements in ACR response rates and disease activity scores, both as monotherapy and in combination with csDMARDs. Infections, particularly herpes zoster, were the most frequent adverse events. The drug's ability to modulate multiple cytokines central to T-cell function explains its robust efficacy in RA [110].

JAK inhibitors represent potent and convenient oral alternatives to biologics, offering multi-cytokine targeting and rapid symptom relief. Differences in JAK isoform selectivity (e.g., JAK1 versus pan-JAK) may influence efficacy and safety, though head-to-head trials are largely lacking. Variations in selectivity and potency across studies make direct comparisons of JAK inhibitors difficult. Highly selective inhibitors offer more predictable responses and fewer off-target side effects, whereas broader inhibitors may enhance efficacy but carry a higher risk of adverse effects [111].

#### 7.1.3 Biological synthetic DMARDs

##### 7.1.3.1 IL-1 inhibitors

Interleukin-1 (IL-1) is a proinflammatory cytokine implicated in the pathogenesis of numerous autoimmune and autoinflammatory disorders by triggering an extensive array of downstream cascade events [112].

###### a Anakinra

Anakinra is a recombinant IL-1 receptor antagonist that competitively inhibits IL-1α and IL-1β binding. It is approved by the FDA for the treatment of severe rheumatoid arthritis (RA) in adults, as well as for rare autoinflammatory conditions such as Neonatal-Onset Multisystem Inflammatory Disease (NOMID) and Deficiency of the IL-1 Receptor Antagonist (DIRA). Anakinra provides a steroid-sparing effect and offers several advantages, including a short half-life (<24 h), which allows flexible dose adjustment and minimizes long-term toxicity [113].

Although its approved indications are limited, off-label use has shown efficacy in a wide spectrum of inflammatory and rheumatologic diseases, including Multisystem Inflammatory Syndrome in Children (MIS-C), Kawasaki disease (KD), and polyarticular Juvenile Idiopathic Arthritis [4]. The Paediatric Rheumatology community has highlighted IL-1 blockade as both safe and effective in autoinflammatory disorders [114]. During the COVID-19 pandemic, anakinra also received emergency FDA approval for severe SARS-CoV-2–related hyperinflammation, further supporting rgency FDA approval for severe SARS-CoV-2–related hyperinflammation, further supporting its favourable safety profile. In RA, anakinra demonstrates moderate efficacy in patients with inadequate responses to conventional DMARDs. However, TNF inhibitors such as adalimumab, etanercept, and infliximab generally achieve superior clinical outcomes. Key limitations of anakinra include frequent injection-site reactions and the requirement for daily administration, which reduce patient adherence compared with other biologics [115].

###### b Canakinumab

Canakinumab is a fully human monoclonal antibody that selectively neutralizes IL-1β, thereby preventing receptor-mediated signalling. It has a long plasma half-life (3–4 weeks) and is approved for cryopyrin-associated periodic syndromes (CAPS), systemic Juvenile Idiopathic Arthritis (SJIA), and gouty arthritis [116]. Clinical trials have demonstrated rapid, sustained disease control in CAPS, with normalization of inflammatory biomarkers (CRP, SAA, IL-6) and prevention of disease flares. A pharmacokinetic–flare probability model supports a standard regimen of 150 mg subcutaneously every 8 weeks, which has been validated in randomized trials: none of the canakinumab-treated CAPS patienteated CAPS patients relapsed within 24 weeks, compared with 81 % of placebo-treated patients. Its extended half-life allows less frequent dosing compared with anakinra, offering greater convenience and adherence. Long-term safety data indicate good tolerability, with only mild injection-site reactions, slightly increased non-serious infections, and no evidence of anti-drug antibody development. Pharmacokinetics are dose-proportional and consistent across age, sex, and disease state [117]. IL-1 inhibitors represent important therapeutic options, though their role in RA remains limited compared to TNF-α or IL-6 blockade, and ongoing research is needed to identify patient subsets most likely to benefit.

##### 7.1.3.2 IL-6 inhibitors

Interleukin-6 (IL-6) plays a key role in RA by driving B-cell differentiation, antibody production, Th17 cell development, release proinflammatory cytokines and chemokines. Excess IL-6 contributes to systemic inflammation and chronic disease progression. Therapeutic blockade of IL-6 or its receptor (IL-6R) has therefore become an established treatment strategy in RA [118].

###### a Tocilizumab (TCZ)

TCZ a humanized IgG1 monoclonal antibody that binds to both soluble and membrane-bound IL-6R, thereby prevents IL-6 from activating downstream signalling pathways. It is available in both intravenous and subcutaneous formulations and was the first IL-6R inhibitor approved for RA. It demonstrated significant efficacy as monotherapy or in combination with csDMARDs, including in methotrexate-naïve patients. Clinical benefits include reduced disease activity, improved physical function, and normalization of acute-phase reactants such as CRP. Safety concerns mainly involve increased infection risk, neutropenia, elevated liver enzymes, and hyperlipidaemia [[119], [120], [121]].

###### b Sarilumab

Sarilumab is a fully human IgG1 monoclonal antibody targeting both soluble and membrane-bound IL-6R with high affinity, effectively blocking both classical and trans signalling pathways. Approved by the FDA in 2017 for patients with moderate-to-severe RA refractory to DMARDs, it is administered subcutaneously every two weeks. Clinical trials demonstrated robust efficacy, including in patients with inadequate response to TNF inhibitors. The safety profile is broadly comparable to TCZ, with infections, neutropenia, liver enzyme elevations, and hyperlipidaemia being the most frequently observed adverse events [[122], [123], [124]]. IL-6R inhibitors represent an effective therapeutic option in RA, particularly for patients requiring biologic monotherapy or those who fail TNF blockade. Ongoing research is investigating selective inhibition of IL-6 trans signalling to refine therapeutic efficacy while minimizing adverse events.

##### 7.1.3.3 TNF-α blockers

Tumor necrosis factor-alpha (TNF-α) is a pivotal proinflammatory cytokine in RA pathogenesis. Primarily produced by macrophages and Th1 cells, TNF-α activates synovial fibroblasts, drives chemokine release, recruits inflammatory cells, and induces matrix metalloproteinases (MMPs), ultimately causing cartilage degradation and bone erosion [125,126]. Currently, five TNF inhibitors are licensed for RA: four administered subcutaneously (adalimumab, certolizumab pegol, etanercept, golimumab) and one intravenously (infliximab) [127].

###### a Infliximab

Infliximab is a chimeric monoclonal antibody (mouse variable region, human IgG1) that binds both soluble and membrane-bound TNF-α with high affinity, preventing receptor activation and downstream inflammatory signalling. In RA, infliximab is most effective when combined with methotrexate, which enhances drug persistence and reduces the formation of anti-drug antibodies [128]. Clinical trials demonstrated significant improvements in ACR20 and DAS28 remission with infliximab plus MTX at 3 mg/kg, with no added efficacy at 10 mg/kg but higher infection risk, including tuberculosis. Accordingly, careful TB screening is essential, and routine high-dose induction is not recommended [129].

###### b Adalimumab

Adalimumab is a fully human, recombinant monoclonal antibody. It binds to tumor necrosis factor-alpha (TNF-α) and blocks its interaction with TNFR1 and TNFR2 receptors. This prevents further inflammatory signals, reducing synovial inflammation, joint damage, and overall symptoms of rheumatoid arthritis [130]. Administered subcutaneously (40 mg every other week), it has shown robust efficacy in major trials (ARMADA, DE019, STAR), both as monotherapy and in combination with MTX. Benefits include rapid onset (week 1), sustained responses up to 5 years, slowed radiographic progression, and improved health-related quality of life. Safety is generally favourable, with injection-site reactions and mild cutaneous events most common [131].

###### c Golimumab

Golimumab (GLM) is a humanized IgG1κ antibody monoclonal that got FDA approval in 2009 and is marketed under the brand name Simponi. It is made from a murine hybridoma cell line. It has been used to treat axial spondylarthritis, psoriatic arthritis, polyarticular juvenile idiopathic arthritis, and rheumatoid arthritis [132]. Administered monthly subcutaneously, it has demonstrated efficacy in RA patients with inadequate response to prior TNFis, maintaining low disease activity in up to 50 % of patients at 18 months. Real-world studies show high short-term adherence (>85 %) but reduced long-term persistence in RA compared to spondylarthritis [133]. Its safety profile is consistent with other TNFis, with no new safety signals identified [134,135].

###### d Etanercept

Etanercept is a specific anti-cytokine drug used in the management of rheumatoid arthritis (RA). Structurally, it consists of a 75 kDa tumor necrosis factor receptor II fused to the Fc portion of human IgG1, a design that influences its immunogenicity, persistence, and infection risk [136]. In RA, etanercept plus MTX yields superior outcomes versus MTX alone, with higher ACR responses and slower radiographic progression [137]. Biosimilars (e.g., SB4, LBEC0101, CT-P13) have shown equivalent efficacy, safety, and immunogenicity, with some reporting fewer injection-site reactions and lower anti-drug antibody rates [138,139].

###### e Certolizumab pegol

Certolizumab pegol (CZP) is a PEGylated Fab' fragment of a humanized monoclonal antibody that targets TNF-α. It has a molecular weight of around 90.8 kDa and a dissociation constant of approximately 90 pM. The absence of an Fc region in CZP prevents complement fixation, antibody-dependent cytotoxicity, and the activation of apoptosis, in contrast to other TNF inhibitors. With no cross-reactivity to TNF-β or healthy human tissues, it efficiently neutralizes both soluble and membrane-bound TNF-α in a dose-dependent manner and prevents monocytes from producing TNF-α and IL-1β in response to LPS. CZP differs from other TNF blockers due to its Fc-free structure, which reduces immune-mediated cytotoxicity and restricts placental transfer [140].This structural feature also minimizes placental transfer, making CZP uniquely suitable for women of reproductive age. Clinical trials (RAPID 1, REALISTIC) demonstrated rapid, sustained improvements in disease activity, radiographic progression, and patient-reported outcomes, with efficacy maintained in 5-year extensions [[141], [142], [143]]. The CRIB study confirmed negligible fetal exposure and no increase in adverse pregnancy outcomes, supporting its use during pregnancy [144].

**Fig. 6:**
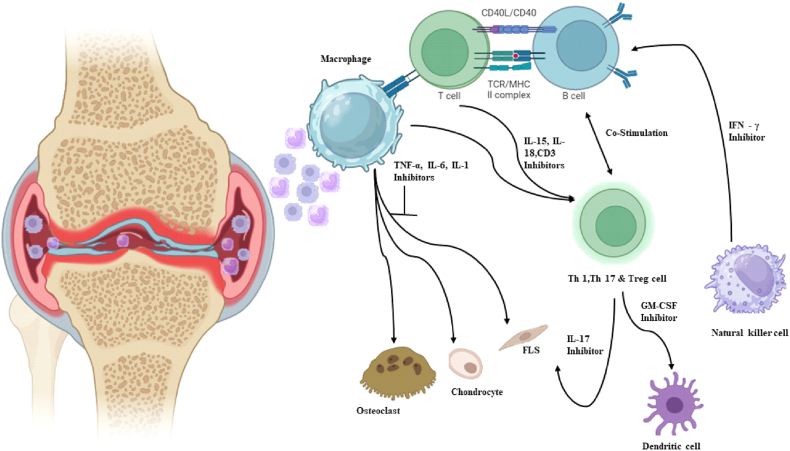
Targeted immune pathways and therapeutic interventions in rheumatoid arthritis.

TNF inhibitors remain a cornerstone of RA management, with extensive long-term safety and efficacy data supporting their use following conventional synthetic DMARD failure. Despite their success, up to half of patients discontinue TNF inhibitors within 3 years, underscoring the need for alternative biologics and targeted therapies [145] (Fig. 6).

### 7.2 CAR-T cellular therapy in RA

Chimeric antigen receptor (CAR) T cell therapy is a novel immunotherapy approach in which a patient's T cells are genetically engineered to express CARs that recognize specific antigens, enabling precise targeting and elimination of pathogenic cells. While initially developed for hematologic malignancies, CAR-T therapy is now being investigated for autoimmune diseases such as rheumatoid arthritis (RA), where chronic inflammation and joint damage are driven by autoreactive B and T cells [146,147].

Conventional T cells recognize foreign versus self-peptide–MHC complexes (pMHCs) via their T-cell receptors (TCRs), requiring high-affinity agonist pMHCs for activation. CARs are bioengineered receptors that bypass MHC restriction, allowing T cells to target specific antigens independently. They combine antibody-like antigen specificity with T-cell proliferation and cytotoxic activity, recognizing proteins, glycolipids, and carbohydrates. MHC-independent recognition also allows CAR-T cells to evade mechanisms such as HLA downregulation. Structurally, CARs consist of: Single-chain variable fragment (scFv), Hinge/spacer domain, Transmembrane α-helix, Intracellular signalling domains (ITAMs). The scFv and intracellular domains drive antigen recognition and T-cell activation, while the transmembrane and spacer domains fine-tune function. In the context of RA, CAR-T therapy aims to selectively target autoreactive lymphocytes and modulate inflammatory cytokines, offering a precise and potentially disease-modifying intervention [148].

#### 7.2.1 Axicabtagene ciloleucel (axi-cel)

Axicabtagene ciloleucel is an autologous anti-CD19 CAR T-cell therapy developed for patients with relapsed or refractory B-cell malignancies, particularly diffuse large B-cell lymphoma. In this therapy, patient-derived T cells are genetically engineered to express a chimeric antigen receptor (CAR) targeting CD19 on B cells. Binding to CD19 activates the intracellular CD28 and CD3ζ domains, inducing T-cell proliferation, cytokine secretion, and cytolytic killing of CD19-positive cells. The persistence of CAR T cells in circulation contributes to durable antitumor responses. Regulatory approval was based on the ZUMA-1 phase II trial. Among 101 patients with relapsed or refractory diffuse large B-cell lymphoma, primary mediastinal B-cell lymphoma (PMBCL), or transformed follicular lymphoma, the objective response rate (ORR) was 83 %, with 58 % achieving complete responses. Median duration of response was 11.1 months, while median overall survival was not reached at 2-year follow-up. The potent immune activation of axi-cel can result in adverse events such as cytokine release syndrome (CRS) and immune effector cell–associated neurotoxicity syndrome (ICANS). Overall, axi-cel provides rapid and durable responses in heavily pretreated diffuse large B-cell lymphoma patients, representing a significant advance in immunotherapy. Despite the potential for severe toxicities, careful monitoring and prompt intervention allow safe use in eligible patients [149].

#### 7.2.2 Tisagenlecleucel

Tisagenlecleucel is a CD19-directed autologous CAR-T therapy approved for relapsed/refractory (r/r) paediatric and young adult acute lymphoblastic leukemia (ALL), diffuse large B-cell lymphoma (DLBCL), and follicular lymphoma (FL). It is available in over 34 countries across more than 430 certified centres, with over 7000 patients treated in clinical trials and real-world settings. The most common toxicity is cytokine release syndrome (CRS), which is generally managed effectively with tocilizumab following standardized ASTCT grading. Immune effector cell–associated neurotoxicity syndrome (ICANS) can occur, presenting as headache, seizures, or delirium, and typically resolves with supportive care. Other potential risks include infections, tumor lysis syndrome, and prolonged B-cell aplasia, often managed with prophylactic immunoglobulin therapy. Replication-competent lentivirus remains a theoretical risk but has not been observed clinically. CAR-T cell expansion correlates with clinical response, particularly in ALL compared to DLBCL. B-cell aplasia serves as a functional marker of CAR-T persistence, and early B-cell recovery (<6 months) may indicate inferior outcomes. Minimal residual disease (MRD) monitoring using next-generation sequencing predicts relapse more sensitively than B-cell recovery alone. Tisagenlecleucel exemplifies how CAR-T therapies can provide durable remissions in heavily pretreated hematologic malignancies [150].

#### 7.2.3 Brexucabtagene autoleucel

Brexucabtagene autoleucel (brexu-cel) is an autologous CD19-directed chimeric antigen receptor (CAR) T-cell therapy approved for relapsed or refractory (R/R) mantle cell lymphoma (MCL). It is a CD28-based CAR-T therapy, in contrast to tisagenlecleucel, which utilizes a 4-1BB costimulatory domain. Brexu-cel is approved in the United States, European Union, and other regions for R/R MCL, as well as for R/R B-cell acute lymphoblastic leukemia (B-ALL).

Structurally, brexu-cel is essentially the same CAR-T construct as axicabtagene ciloleucel (axi-cel), approved for R/R B-cell non-Hodgkin lymphomas, with an additional processing step to remove circulating blasts during leukapheresis. Approval was based on the pivotal phase II ZUMA-2 trial, which demonstrated an objective response rate (ORR) of 91 % and a complete response (CR) rate of 68 %. Median duration of response (DOR) was 28.2 months, median progression-free survival (PFS) was 25.8 months, and median overall survival (OS) was 46.6 months [151].

#### 7.2.4 Lisocabtagene maraleucel (Liso-cel)

Lisocabtagene maraleucel is an autologous CD19-directed CAR-T cell therapy that incorporates a 4-1BB costimulatory domain. Unlike earlier constructs, Liso-cel is manufactured using a defined ratio of CD4^+^ and CD8^+^ T cells to enhance functional synergy and reduce variability in clinical responses. The pivotal TRANSCEND NHL 001 phase I trial evaluated Liso-cel in patients with relapsed/refractory (R/R) B-cell non-Hodgkin lymphomas, including diffuse large B-cell lymphoma (DLBCL), follicular lymphoma (FL), chronic lymphocytic leukemia (CLL), marginal zone lymphoma, and primary mediastinal B-cell lymphoma [152]. Subsequent pivotal trials (TRANSCEND, TRANSFORM, PILOT) confirmed the efficacy and safety of Liso-cel, leading to regulatory approval for R/R large B-cell lymphoma (LBCL). The cohort included heavily pretreated patients (median three prior therapies), many with high-risk features such as advanced age, comorbidities, CNS involvement, or organ dysfunction—approximately one-third of whom would not have met clinical trial eligibility criteria. The incidence of grade ≥3 cytokine release syndrome (CRS) and immune effector cell–associated neurotoxicity syndrome (ICANS) was similar to clinical trial reports, despite the inclusion of high-risk patients. Proactive and more frequent use of tocilizumab and corticosteroids did not compromise efficacy and may have facilitated broader applicability in medically complex populations [153].

#### 7.2.5 Idecabtagene vicleucel

Idecabtagene vicleucel (Ide-cel) is an autologous B-cell maturation antigen (BCMA)–directed CAR-T cell therapy, approved in March 2021 by the U.S. Food and Drug Administration (FDA) for the treatment of adult patients with relapsed/refractory multiple myeloma (RRMM) after at least four prior lines of therapy, including a proteasome inhibitor, an immunomodulatory agent (IMiD), and an anti-CD38 antibody. Approval was based on the pivotal KarMMa phase II trial, which demonstrated clinically meaningful outcomes in a heavily pretreated population. In this approach, patient-derived T cells are transduced with a lentiviral vector encoding a CAR composed of a murine single-chain variable fragment (scFv), a CD8α hinge and transmembrane region, and 4-1BB/CD3ζ signaling domains. Engagement of BCMA by the CAR triggers T-cell activation, proliferation, cytokine secretion, and cytolytic killing of malignant plasma cells. Neurotoxicity was observed and generally managed with corticosteroids and antiseizure medications. Hemophagocytic lymphohistiocytosis/macrophage activation syndrome (HLH/MAS) occurred in 4 % of patients, including two fatalities. Prolonged cytopenias were common, additional risks included hypogammaglobulinemia and infections. Given these risks, Ide-cel carries boxed warnings for CRS, neurotoxicity, HLH/MAS, and prolonged cytopenia [154].

#### 7.2.6 Ciltacabtagene autoleuce

Ciltacabtagene autoleucel (cilta-cel) is a second-generation autologous CAR-T cell therapy directed against B-cell maturation antigen (BCMA). It incorporates two single-chain variable fragments (scFvs) that bind distinct BCMA epitopes, a transmembrane domain, and intracellular CD3ζ and 4-1BB costimulatory signaling domains. Upon binding BCMA on malignant plasma cells, cilta-cel activates T cells, leading to cytokine release and cytotoxic lysis via perforin–granzyme and Fas–FasL pathways. BCMA is an attractive therapeutic target because it is expressed on plasmablasts, plasma cells, and malignant plasma cells, but absent on naïve/memory B cells and hematopoietic stem cells. Cilta-cel received FDA approval on February 28, 2022, for the treatment of relapsed/refractory MM after ≥3 prior lines of therapy.

Adverse events are consistent with other CAR-T therapies include Cytokine release syndrome (CRS), Neurotoxicity, Neutropenia, anemia, and thrombocytopenia were common. Most deaths were disease-related; treatment-associated fatalities remain rare. Long-term safety monitoring is mandated post-approval, and trials are exploring its potential as a first-line CAR-T therapy in MM [155].

Therapeutic targeting of T cells has significantly enhanced the therapy of rheumatoid arthritis, surpassing the clinical alleviation offered by NSAIDs and corticosteroids. DMARDs, including conventional (csDMARDs), targeted synthetic (tsDMARDs), and biologic (bDMARDs), have altered the course of treatment by modifying T-cell activation, cytokine signaling, and immune-mediated joint damage. While bDMARDs provide targeted blocking of cytokines such as TNF-α, IL-1, and IL-6, and tsDMARDs such as JAK inhibitors allow for multi-cytokine regulation with oral convenience, csDMARDs such as methotrexate, hydroxychloroquine, and sulfasalazine remain the mainstay of treatment. Despite these advancements, problems like uneven patient reactions, safety concerns, and partial remission persist. CAR-T cell therapy and other cellular immunotherapies, in particular, offer the potential to produce long-term remission or even cure while maintaining protective immunity by selectively decreasing pathogenic T and B cell subsets.

## 8 Targeted T cell therapy in clinical trials

In August 2024, a thorough search was conducted using the clinicaltrials.gov database (https://clinicaltrials.gov/) to identify studies on "rheumatoid arthritis.” The search was refined using relevant keywords such as T cell therapy AND Rheumatoid Arthritis. Studies that treated unrelated conditions, such as Sjögren's syndrome, cancer, Epstein-Barr virus-associated viremia, or refractory autoimmune diseases, were excluded. For each relevant trial, detailed information about the medications used and study parameters was gathered. The expected outcomes in each category were evaluated, and the number of trials testing the efficacy of T cell-based therapies was recorded and tabulated in Table 2.

**Table 2:** Clinical trials of cellular and immune-targeted therapies in rheumatoid arthritis (RA).

NCT	Phase/Study type	Intervention	Target	Primary outcome	Secondary outcome	Status
NCT06475495	Phase I & II, Interventional (Not yet recruiting)	Anti-CD19 CAR-T (KYV-101) & Rituximab	B-cell depletion in treatment & Anti-citrullinated protein antibody (ACPA) + refractory RA	Phase 1: Incidence & severity (0–4) of CRS, ICANS, AEs, SAEs after CAR-T Phase 2: percentage of ACPA seroconversion (<20 mU/ml)	Drug-free survival, relapse/flare rate, ACR20/50/70 response, DAS28-CRP remission, SDAI remission, Boolean 2.0 remission, changes in RA activity scores, B-cell depletion duration, CAR-T persistence, ACPA/RF changes, immunoglobulin changes	Not yet recruiting
NCT06390709	Observational	Lab Collection Only	JAK inhibitors (tofacitinib, upadacitinib), T-cell inhibitor (abatacept), IL-6 inhibitor (tocilizumab)	Rate of MSRC to predict treatment response (based on CDAI disease activity at 6 months)	–	Recruiting
NCT05782335	Observational	Abatacept (CTLA-4-Ig) vs TNFi/csDMARD controls vs healthy controls	Immune repertoire (TCR & BCR)	Change in clonotype diversity of TCR and BCR repertoires at baseline, 3, and 6 months	CTLA4 gene expression, Changes in T, B, NK cell subsets, Expression of 150 immune response genes, Correlation of repertoire changes with CDAI, SDAI, DAS28, PROs (RA-FQ, MDHAQ)	Recruiting (estimated completion Oct 2025)
NCT03813771	Interventional, Phase IV	Arm A: MTX 15 mg/week PO (+HCQ 400 mg od) Arm B: MTX 15 mg/week PO (+HCQ 400 mg od) Arm C: MTX 15 mg/week PO (+HCQ 400 mg od) in combination with Etanercept (Benepali®)	Naïve CD4^+^ T-cell frequency	Proportion of patients in clinical remission (DAS28-ESR ≤2.6) at 24 weeks (MTX vs MTX + Etanercept arms stratified by T-cell status)	Clinical remission at 12 weeks, Sustained remission (12 & 24 weeks), PROs (EMS, VAS, HAQ-DI), Imaging remission (Doppler synovitis score = 0), Immunological remission (normal naïve CD4^+^ T-cells at 24 wks), Cumulative steroid use	(Unknow) estimated completion 2023
NCT06994143	Interventional, Phase I	CLN-978 (subcutaneous CD19-directed T cell engager)	CD19^+^ B cells (T-cell mediated B-cell depletion)	Incidence and severity of adverse events/serious adverse events over 48 weeks	Serum concentrations of CLN-978 (PK), Anti-drug antibodies (ADA), Levels of circulating B lymphocytes (PD biomarker)	Recruiting (Study Completion Estimated 2028)
NCT07100873	Interventional, Phase I	ADI-001 (Anti-CD20 CAR-T), Fludarabine, Cyclophosphamide	CD20 on B cells via γδ T-cell CAR construct	Incidence of dose-limiting toxicities within each dose level cohort (Safety & tolerability) – 28 days	Clinical responses (RA disease activity), Persistence and expansion of ADI-001 cells, Immunophenotyping and biomarker changes, Longer-term safety (adverse events, infections, etc.)	Not yet recruiting (Study Completion Estimated 2028)
NCT06201416	Interventional, Phase I	Autologous regulatory CAR-Treg therapy (SBT777101), single i.v. infusion	Regulatory T cells (Tregs)	Safety & tolerability: incidence, nature, severity of AEs; dose-limiting toxicities (CRS, ICANS, organ/hematologic toxicity)	PK/PD, Treg persistence & expansion, synovitis changes, clinical activity (DAS28, CDAI), immunologic biomarkers	Recruiting (Study Completion Estimated 2026)
NCT07123038	Observational, Phase IV	Long-Term Safety Monitoring Procedures in patients who exposed to SBT777101 Treg cell therapy	Regulatory T cells (Tregs)	Incidence, type, severity, and causality of delayed adverse events (15 years)	Persistence of gene-modified Tregs, incidence of replication competent lentivirus (RCL), mortality	Recruiting (Study Completion Estimated 2040)

## 9 Current challenges and future prospects

Diagnosing, preventing, and treating RA, as well as forecasting individual treatment responses, still have gaps despite significant therapeutic advancements. To enable precision medicine and provide treatments with homeostatic potential, a deeper comprehension of disease biology is necessary [156]. The therapeutic effect of bDMARDs and tsDMARDs on long-term mortality is yet unknown, despite the fact that they enhance RA symptoms and quality of life. While some studies show no discernible change when compared to csDMARDs, others point to possible advantages through decreased systemic inflammation and glucocorticoid use [157]. Biologics are better than csDMARDs, but remission rates are still low, and many patients don't reach significant goals like functional independence and pain alleviation. There is still a discrepancy between patient-reported results and better disease scores [158]. Small medicines like Janus kinase (JAK) inhibitors and biologic medications like anti-TNF and anti-IL-6 therapies have greatly enhanced outcomes in RA. Patients with severe or refractory diseases are currently the only ones who can use them. Numerous challenges remain, including: (i) a sizable portion of patients do not react favorably to biologics or JAK inhibitors; (ii) long-term safety concerns, including the potential for infection, cardiovascular events, and cancer, are not fully addressed; and (iii) the high cost of biologics continues to limit access in many healthcare settings. JAK inhibitors have efficacy on comparable levels with or even better than biologics, with the added advantages of oral administration and lower production costs; nevertheless, their long-term effects on disease modification, safety, and mortality are still unknown [159]. Accurate targeting is hampered by cell plasticity and overlapping cytokine patterns. Stable and predictable immunological therapies are limited by the absence of accurate markers to differentiate nTregs from iTregs and our poor understanding of transcriptional and epigenetic regulation [160]. Although lymphodepletion increases efficacy, it also increases the danger of infection. This is one of the limits of CAR T and CAR-Treg therapy.B cell aplasia, hypogammaglobulinemia, and persisting pathogenic plasma cells are examples of long-term safety concerns. Inflammatory circumstances may cause FOXP3+ cells to transform. Both T and B cell-driven disease processes cannot be controlled concurrently by current methods without resulting in widespread immunosuppression [161].

The management of RA may be revolutionized by new approaches such as CAR-T and CAR-Treg therapies, which selectively destroy autoreactive cells or create immunological tolerance. However, the heterogeneity of autoreactive lymphocytes, dangers such as cytokine release syndrome, and high prices continue to be obstacles [162]. JAK/STAT inhibitors, which have been improved to lessen adverse effects, provide oral, affordable substitutes for biologics. Long-lasting control may be made possible by vaccination techniques that produce endogenous anti-cytokine antibodies (such as TNFα and IL-23). Although its dual functions need to be clarified, novel targets include IL-32 and IL-34 for suppression and IL-35 for increasing immunological tolerance [163]. Understanding immunological variety, disease causes, and treatment responses will be improved by developments in TCR and BCR repertoire analysis, which are fueled by bulk and single-cell sequencing with sophisticated analytics. Predictive diagnoses, tailored immunotherapies, and treatment outcome monitoring for cancer, infections, and autoimmune diseases may be made possible by integration with machine learning and liquid biopsy techniques [164]. In order to reduce harm and achieve disease control, future RA therapy must produce evidence-based methods for patients with comorbidities. For patients who cannot afford pricey biologics, cost-effective alternatives such as csDMARD optimization, tapering regimens, and biosimilars are essential [165]. Restoring immunological tolerance, streamlining tapering procedures, and determining predictors of long-lasting remission should be the main goals of future strategies. Long-term remission could be one step closer to a functional cure with a better understanding of immune modulation [166].

## 10 Conclusion

T cells play a crucial and intricate role in the pathophysiology of rheumatoid arthritis (RA), together with B cells, macrophages, and other immunological mediators to produce persistent inflammation and joint degeneration. Their activation triggers a cascade of processes, including as cytokine release, autoantibody production, and synovial hyperplasia, that ultimately lead to the loss of bone and cartilage. Activated T cells initiate and differentiate in a variety of immune cells, including B cells and macrophages. The autoimmune response is subsequently reinforced by the proinflammatory cytokines and autoantibodies produced by these cells. These cells differentiation results in inflammation of the joints, which damages the bones, cartilage, and causes synovial hyperplasia. Understanding these systems is crucial since they are important nodes for therapeutic intervention. The better clinical outcomes of existing drugs that target T cell-mediated pathways, like cytokine inhibition, have demonstrated the therapeutic significance of T cells in RA. However, T cell plasticity, instability of regulatory subsets, and overlapping functions across lineages continue to complicate effective targeting. Future research must focus on understanding the transcriptional and epigenetic mechanisms governing T cell plasticity, identifying biomarkers to predict treatment response, and developing strategies to restore immunological tolerance over the long term. Advancements in these areas could lead to more precise, tailored treatment plans, ultimately aiming for long-term RA remission or perhaps a functional cure.

## CRediT authorship contribution statement

**Monisha Anandan:** Writing – original draft, Data curation, Conceptualization. **J. Narayanan:** Writing – review & editing, Visualization, Supervision.

## Funding information

This work did not receive any specific grant from funding agencies in the public, commercial, or not-for-profit sectors.

## Declaration of competing interest

The authors declare that they have no known competing financial interests or personal relationships that could have appeared to influence the work reported in this paper.

## Data Availability

No data was used for the research described in the article.

## **Glossary**

RA -: Rheumatoid arthritis
CD4^+^ T: Clusters of differentiation 4 or Helper T cell
TNF-α –: Tumor Necrosis Factor -Alpha
IL-1: Interleukin 1
IL-2: Interleukin 2
IL-17: Interleukin 17
Th 1: T-helper cell 1
Th 2 -: T-helper cell 2
Th17: T-helper cell 17
Th9: T-helper cell 9
APCs –: Antigen Presenting Cells
TCR: T cell receptor
NSAIDS: Non Steroidal Anti Inflammatory Drugs
DMARDS: Disease Modifying Antirheumatic Drugs
JAK: Janus kinases
CART -: Chimeric antigen receptors T cells
TGF-β-: Transforming Growth Factor Beta
tTregs: Thymus Tregs
pTregs –: Peripherally Tregs
iTregs: Induced Tregs
GM-CSF -: Granulocyte-macrophage colony-stimulating factor
STAT5 -: Signal Transducer and Activator of Transcription 5
TLR: Toll Like receptor
IgE -: Immunoglobulin E
IgG -: Immunoglobulin G
RAR -: Retinoic acid receptor
(ROR) γt -: Retinoic Acid Receptor-Related Orphan Receptor
DCs -: Dendritic cells
M1 and M2: Macrophage 1 & 2
C-X-C-: Chemokine motif ligand 1
BCR: B Cell Receptor
NBCs: Naive B cells
Tfh -: Follicular helper cells
Tph: Peripheral helper cells
IFN-γ-: Interferon Gamma
RASFs -: Rheumatoid Arthritis Stem Cells
SFs: Synovial fluids
PD-1 -: Programmed cell death protein 1
NK cells: Natural Killer cells
ITIM: Immunoreceptor Tyrosine Based Inhibitory Motif
ITSM: Immunoreceptor Tyrosine Based Switch Motif
RANKL -: Receptor Activator of NF-κB
DAS28: Disease activity score 28
HAQ: Health Assessment Questionnaire
ILD -: Interstitial Lung Disease
FoxP3 -: Forkhead Family Transcription Factor
Gal-9 -: Galectin-9
GSDMD -: Gasdermin D
CIA: Collagen-induced arthritis
mRNA: Messenger RNA
MAPK: Mitogen-activated protein kinase
NF-HEV -: Nuclear Factor Human High Endothelial Venules
VEFG: Vascular Endothelial Growth Factor
CCL17 -: C–C Motif Chemokine Ligand 17
FLS: Fibroblast-like synoviocytes
MMP: Matrix metalloproteinase
MSRC -: Molecular signature response classifiers
FCGR3A -: Fc Gamma Receptor IIIa
ACR50 & ACR70 -: American College of Radiology
ICANS -: Immune Cell Associated Neurotoxicity Syndrome
CRP: C- Reactive Protein
SDAI -: Simple Disease Activity Index
CDAI -: Clinical Disease Activity Index
ACPA -: Anti-citrullinated protein antibody
CTLA-4 -: Cytotoxic T-lymphocyte associated protein 4
CDAI: Crohn's Disease Activity Index
HLA-DRB1: HLA class II histocompatibility antigen
SLE: Systemic Lupus Erythematosus
MD-HAQ: Multidimensional Health Assessment Questionnaire
DLT: Dose Limiting Toxicities
BCMA -: B-cell maturation antigen
MTX: Methotrexate
MHC -: Major histocompatibility complex
HCQ: Hydroxychloroquine
FDA: Food and Drug Administration
TB: Tuberculosis
ADA -: Adalimumab
TOF -: Tofacitinib
OPAL: Optimising Patient Outcomes in Australian Rheumatology
GLM: Golimumab
TCZ –: Tocilizumab
DANCER: Dose Ranging Assessment International Clinical Evaluation of Rituximab
SERENE: Study Evaluating Rituximab's Efficacy in MTX Inadequate Responders
IBD: Inflammatory Bowel Disease
ILD: Interstitial Lung disease
sPDL1: Soluble programmed death ligand 1
